# Supplementary material for: Predicting nodal influence via local iterative metrics
Source: Sci Rep. 2024 Feb 28;14:4929. doi: 10.1038/s41598-024-55547-y (PMC10901818; doi:10.1038/s41598-024-55547-y)
Supplement: Supplementary file 1 — Supplementary Information. [file 41598_2024_55547_MOESM1_ESM.pdf]

Supplementary Information: Predicting Nodal Influence via Local Iterative Metrics

Shilun Zhang[1], Alan Hanjalic[1], and Huijuan Wang[1,*]

[1]Faculty of Electrical Engineering, Mathematics, and Computer Science, Delft University of Technology, Mekelweg 4, 2628 CD, Delft, The Netherlands
[*]H.Wang@tudelft.nl

S1 Computation of an iterative metric set and complexity analysis

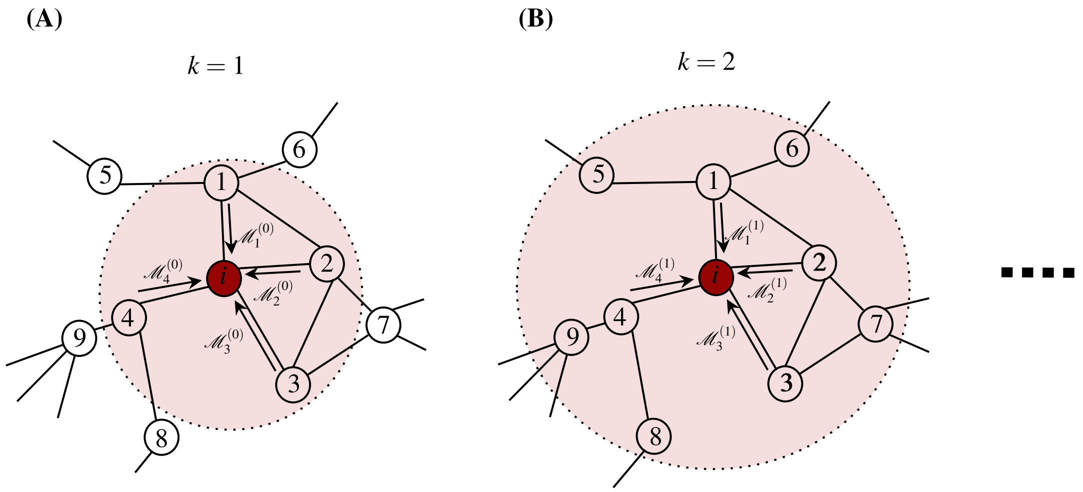

Figure S1. An illustration of the neighborhood considered to compute an iterative metric of order k at any node i.

Here, we exemplify how gradually more network information around a node is incorporated in the computation an iterative metric of a higher order. In each iteration k of an iterative process, the computation of an iterative metric $\mathscr{M}_i^{(k)}$ of node i is derived via aggregating the metrics of its 1-hop neighbors derived in the previous iteration $k-1$. Take the example of NWC computation in the network shown in Figure S1. The iterative process of NWC is governed by $w^{(k)} = Aw^{(k-1)}/|Aw^{(k-1)}|$. Initially, $\mathscr{M}_i^{(0)} = 1/\sqrt{N}$ for any node i, which encode no topological information. In iteration $k=1$, the metric $\mathscr{M}_i^{(1)}$ is derived as the sum of the metrics of its neighbors from iteration 0, i.e., $\mathscr{M}_i^{(1)} = \sum_{j\in\mathscr{N}(i)} \mathscr{M}_j^{(0)}$, where node i's 1-hop neighbor set $\mathscr{N}(i) = \{1,2,3,4\}$. Thus, $\mathscr{M}_i^{(1)}$ encodes the information of 1-hop neighborhood of node i as illustrated in the pink area in Figure S1 (A). In iteration $k=2$, the metric $\mathscr{M}_i^{(2)}$ is derived as $\mathscr{M}_i^{(2)} = \sum_{j\in\mathscr{N}(i)} \mathscr{M}_j^{(1)} = 4\mathscr{M}_i^{(0)} + \mathscr{M}_5^{(0)} + \mathscr{M}_6^{(0)} + 2\mathscr{M}_7^{(0)} + \mathscr{M}_8^{(0)} + \mathscr{M}_9^{(0)}$, which encodes up to 2-hop neighborhood information of node i as illustrated in the pink area in Figure S1 (B). Consequently, for any iteration k, the metric $\mathscr{M}_i^{(k)}$ of node i encodes information of up to k-hop neighborhood.

S2 Training of regression models

Given a network G, we train a Random Forest regression (RFR) model based on the $q = 10\%$ of nodes whose nodal influences are known and use the trained model to predict the influence of the rest nodes. We adopt `RandomForestRegressor` in the Python library `scikit-learn` to implement our regression models. The hyperparameters are determined via 5-fold cross-validation on the training set, where the number of trees considered in RFR, `n_estimators`, is tuned in the range $[50, 1000]$. The final RFR with the identified hyperparameters is trained on the training set.

Performance of all iterative based models in nodal influence prediction is the average over 50 realizations of training set sampling and model training.

S3 Results for performance of iterative metric based models

Figure S2 and Figure S9 show the Mean Squared Errors, r^2, and Recognition rate of top 10% nodes of the three iterative metric based RFR models in all considered real-world networks and LFR networks, respectively. Figure S6 and Figure **??** show the results of iterative metric based Ridge regression models in all considered real-world networks and LFR networks, respectively.

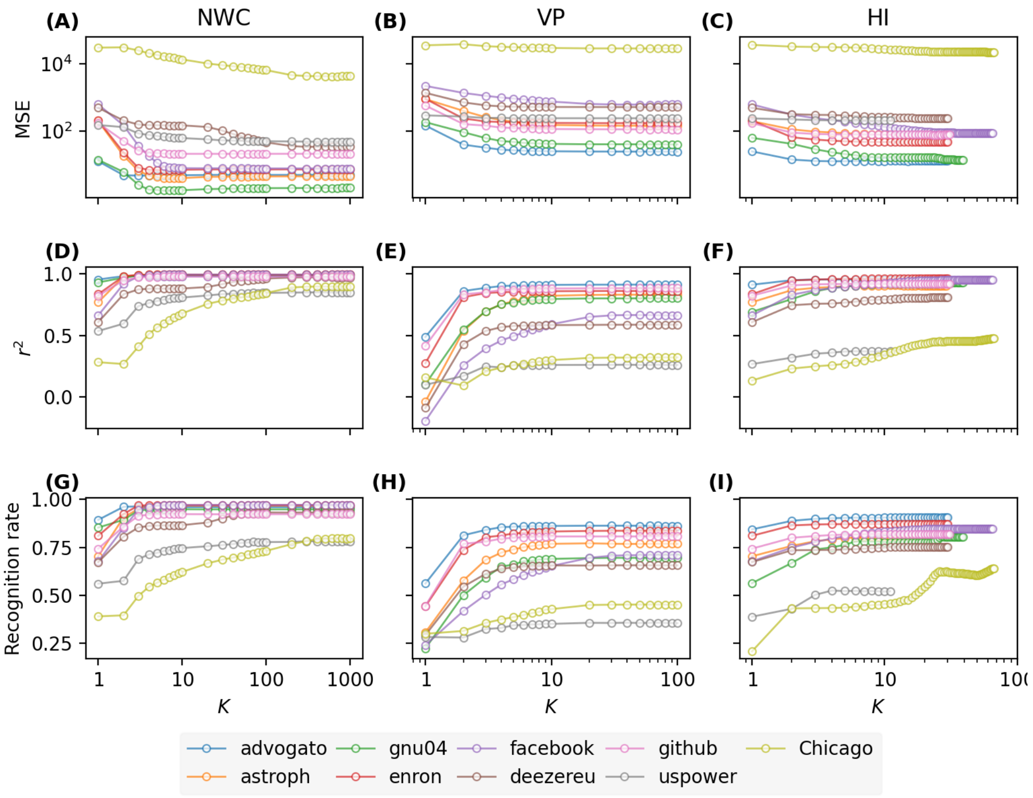

Figure S2. Mean Squared Errors, r^2, and Recognition rate of top 10% nodes of the Normalized Walk Count-, Visiting Probability-, and H index-based models, respectively, in nodal influence prediction as a function of the size K of an iterative metric set in each of the 9 real-world networks.

μ	Diameter	Q	λ_c
0.02	11	0.971	0.060
0.05	8	0.933	0.050
0.1	7	0.859	0.050
0.2	6	0.728	0.050
0.3	6	0.614	0.040
0.4	6	0.497	0.040

Table S1. Basic properties of networks generated by LFR model with 10000 nodes using different mixing parameter μ: network diameter, the modularity Q, epidemic threshold λ_c of the SIR process on the network.

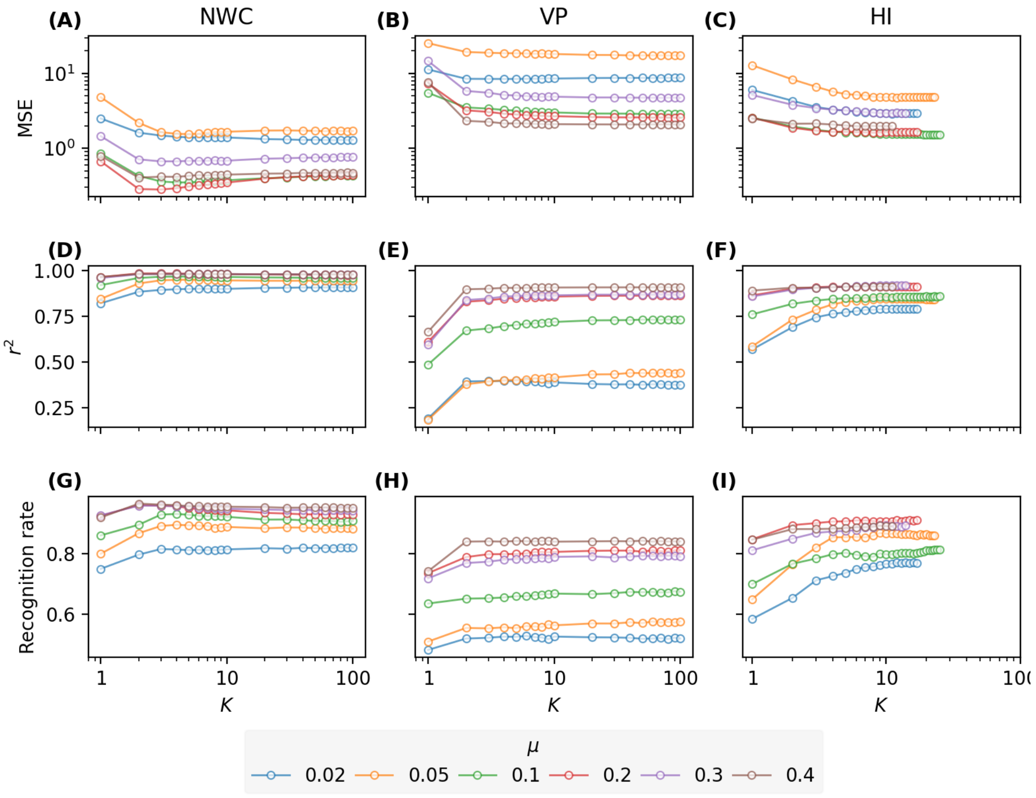

Figure S3. Mean Squared Errors, r^2, and Recognition rate of top 10% nodes of the Normalized Walk Count-, Visiting Probability-, and H index-based models, respectively, in nodal influence prediction as a function of the size K of an iterative metric set in LFR networks with 1000 nodes, where the mixing parameter $\mu = [0.02, 0.05, 0.1, 0.2, 0.3, 0.4]$.

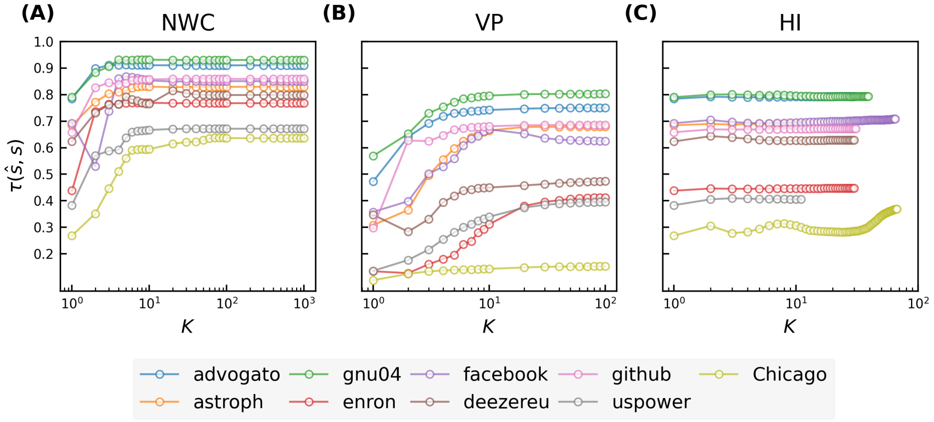

Figure S4. Kendall correlation between the actualy nodal influence s and nodal spreading influence $\hat{s}$ predicted by each iterative metric (NWC, VP or HI) based Ridge regression model as a function of the size K of an iterative metric set in each of the 9 real-world networks.

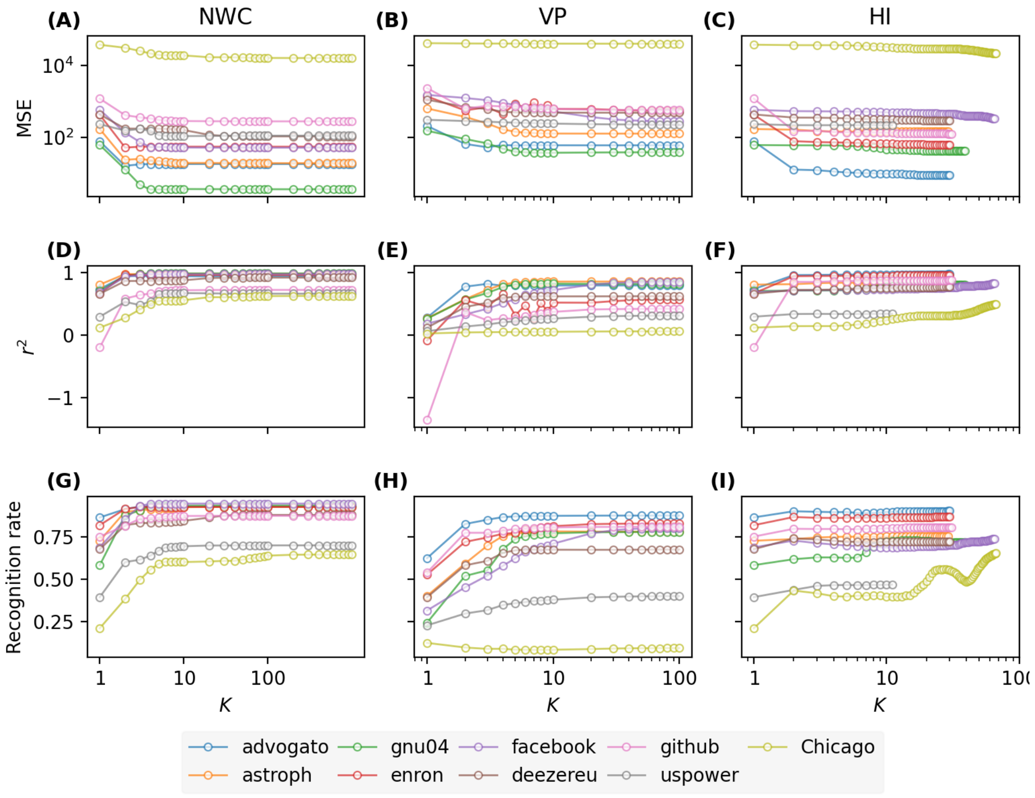

Figure S5. Mean Squared Errors, r^2, and Recognition rate of top 10% nodes of the Normalized Walk Count-, Visiting Probability-, and H index-based Ridge regression models, respectively, in nodal influence prediction as a function of the size K of an iterative metric set in each of the 9 real-world networks.

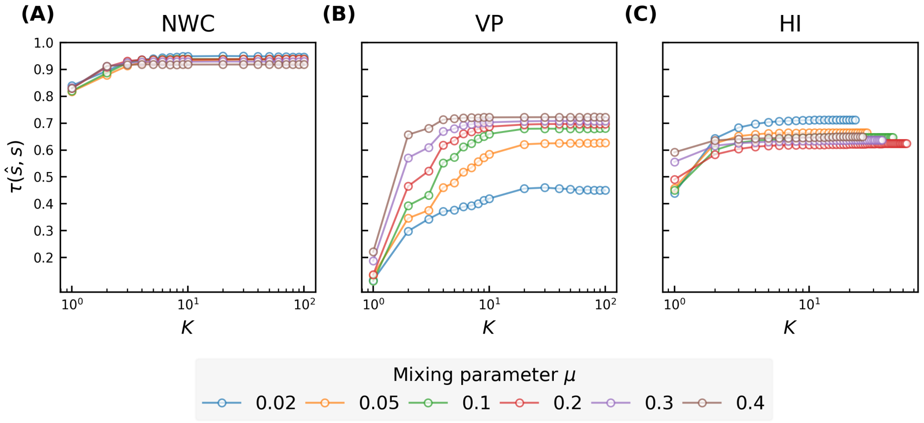

Figure S6. Kendall correlation between the actualy nodal influence s and nodal spreading influence $\hat{s}$ predicted by each iterative metric (NWC, VP or HI) based Random Forest regression model as a function of the size K of an iterative metric set in LFR network with 10000 nodes.

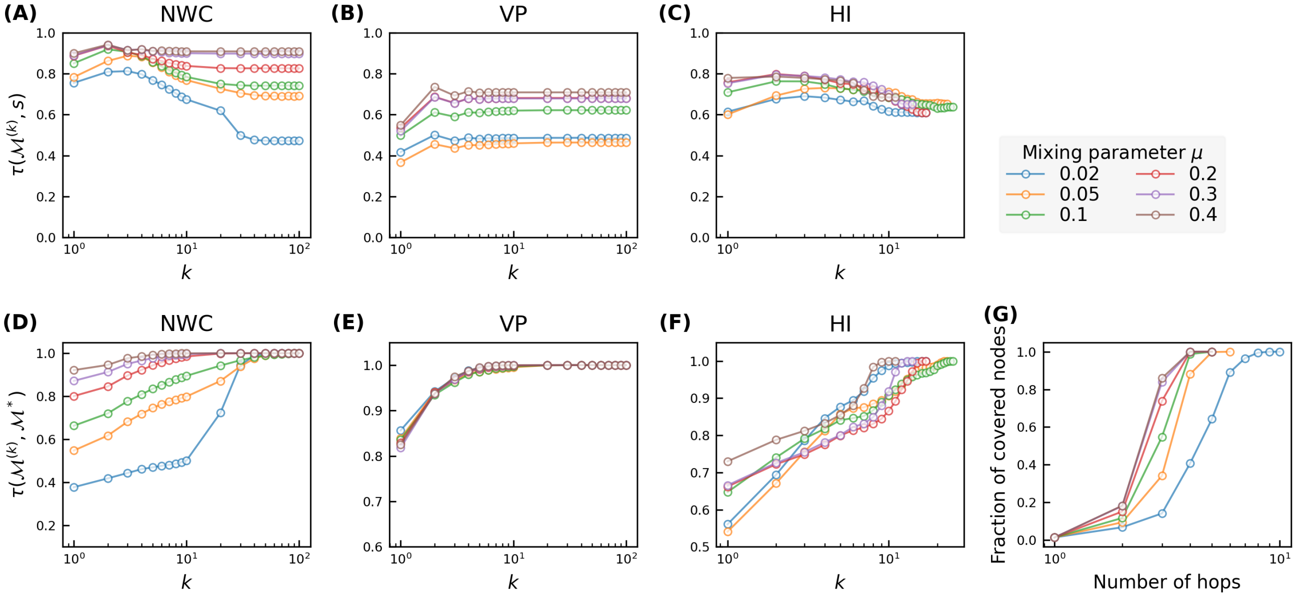

Figure S7. Kendall correlation between nodal spreading influence s and different orders of NWC ($w^{(k)}$, panel A), VP ($p^{(k)}$, panel B), and H index ($h^{(j)}$, panel C), and the convergence of NWC (D), VP (E), HI (F), measured by the Kendall's correlation between the iterative metric after k iterations and the corresponding global centrality metrics, as a function of iteration number k in LFR networks with 10000 nodes and different $\mu = 0.02, 0.05, 0.1, 0.2, 0.3, 0.4$. (G) shows the coverage, i.e. the average fraction of nodes covered by hopping step out from a node, as a function of the number of hops.

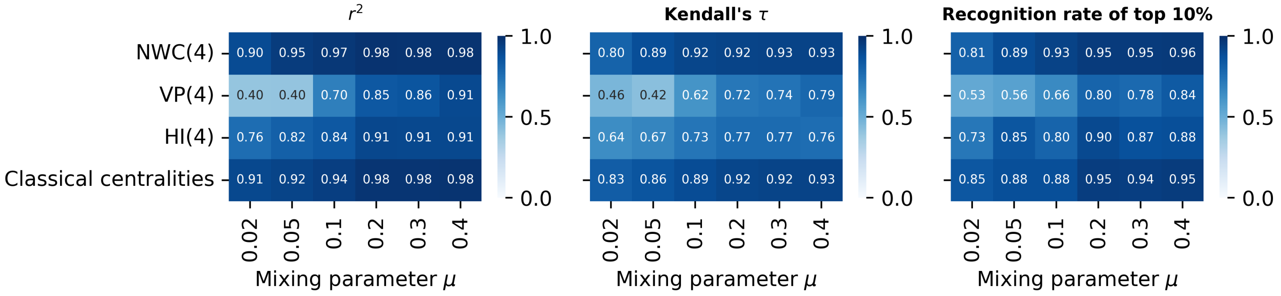

Figure S8. Prediction performance comparison in LFR networks with 10000 nodes of four sets of metrics (vertical axis): Normalized Walk Count when $K = 4$ (NWC(4)), Visiting Probability when $K = 4$ (VP(4)), H index when $K = 4$ (HI(4)), and seven centralities. Three panels correspond to different evaluation measures of predictive models: r^2 (left panel), Kendall's τ (middle panel), and recognition rate of top 10% nodes (right panel), respectively. The prediction quality ratio between NWC based model and the benchmark model ranges from 95% to 106% for all evaluation measures. Results are averaged over 50 realizations of Random Forest Model training process.

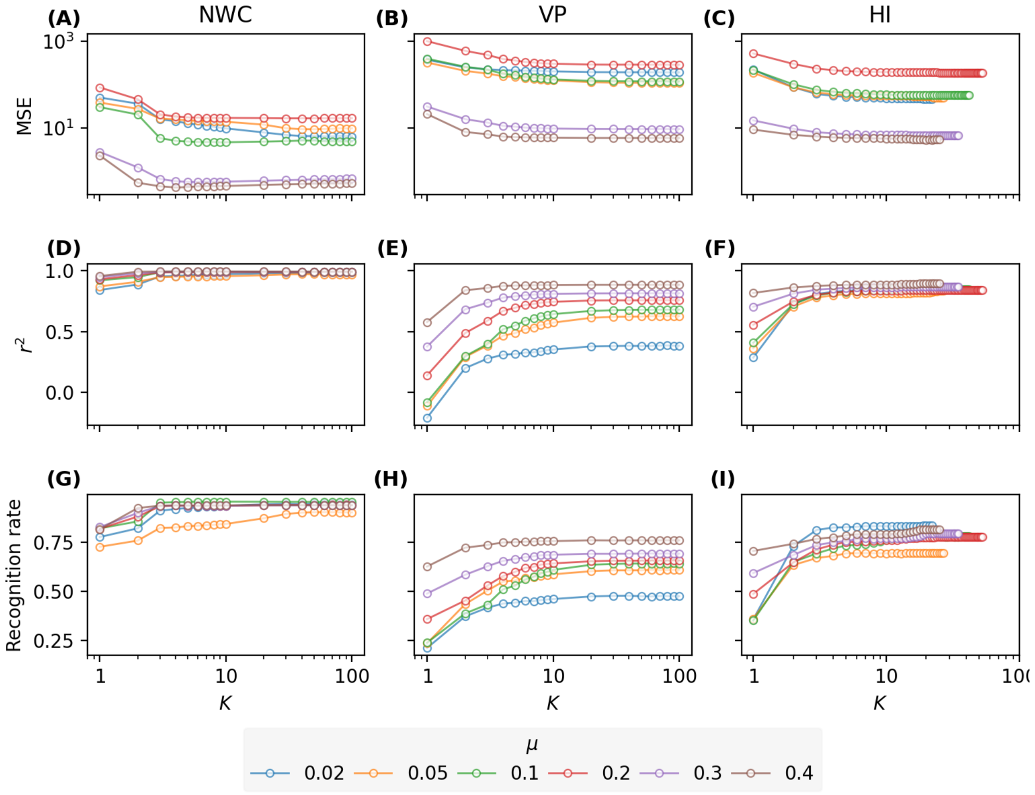

Figure S9. Mean Squared Errors, r^2, and Recognition rate of top 10% nodes of the Normalized Walk Count-, Visiting Probability-, and H index-based models, respectively, in nodal influence prediction as a function of the size K of an iterative metric set in LFR networks with 10000 nodes, where the mixing parameter $\mu = [0.02, 0.05, 0.1, 0.2, 0.3, 0.4]$.
